# Blade-Coating of High Crystallinity Cesium-Formamidinium Perovskite Formulations

**DOI:** 10.1021/acsami.4c04706

**Published:** 2024-07-01

**Authors:** Anaël Jaffrès, Mostafa Othman, Felipe Saenz, Aïcha Hessler-Wyser, Quentin Jeangros, Christophe Ballif, Christian M. Wolff

**Affiliations:** † École Polytechnique Fédérale de Lausanne (EPFL), IEM, PV-Lab, Rue de la Maladière 71b, 2000 Neuchâtel, Switzerland; ‡ Centre Suisse d’Electronique et de Microtechnique (CSEM), Rue Jaquet-Droz 1, 2000 Neuchâtel, Switzerland

**Keywords:** perovskite solar cells, stacking-faults, blade-coating, thiourea, *in situ* monitoring, crystallization

## Abstract

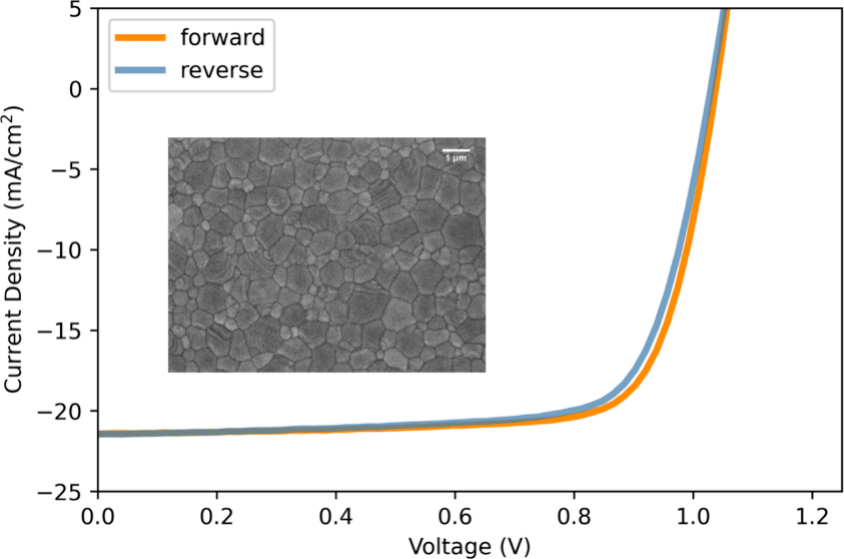

Up-scalable coating processes need to be developed to manufacture efficient and stable perovskite-based solar modules. In this work, we combine two Lewis base additives (N,N′-dimethylpropyleneurea and thiourea) to fabricate high-quality Cs_0.15_FA_0.85_PbI_3_ perovskite films by blade-coating on large areas. Selected-area electron diffraction patterns reveal a minimization of stacking faults in the α-FAPbI_3_ phase for this specific cesium-formamidinium composition in both spin-coated and blade-coated perovskite films, demonstrating its scaling potential. The underlying mechanism of the crystallization process and the specific role of thiourea are characterized by Fourier transform infrared spectroscopy and *in situ* optical absorption, showing clear interaction between thiourea and perovskite precursors and halved film-formation activation energy (from 114 to 49 kJ/mol), which contribute to the obtained specific morphology with the formation of large domain sizes on a short time scale. The blade-coated perovskite solar cells demonstrate a maximum efficiency of approximately 16.9% on an aperture area of 1 cm^2^.

## Introduction

Within a decade, hybrid organic–inorganic metal halide perovskite solar cells (PSCs) have reached efficiencies beyond 25% in single junctions^1,2^ and above 33% in tandem perovskite/Si configurations.^1^ Such developments have been made possible, thanks to the high optoelectronic properties of perovskites, their bandgap tunability, and relatively simple and low-cost fabrication.^3,4^

Formamidinium (FA)-rich perovskites are of special interest due to their enhanced thermal stability with respect to their methylammonium (MA)-rich counterparts^5,6^ and their more favorable bandgaps compared to the fully inorganic CsPbI_3_^7^ (∼1.5 eV for FAPbI_3_ and ∼1.75 eV for CsPbI_3_, on account of the optimal bandgap of 1.33 eV derived from the Shockley-Queisser limit^8^). However, a major drawback of these materials is their poor phase stability, where the cubic photoactive α-phase suffers from a reversible transition to the hexagonal δ-phase (not photoactive) at room temperature.^9^ So far, different strategies have been deployed to stabilize the FAPbI_3_ α-phase: additives,^10^ surface passivation,^11^ solvent engineering,^12^ and compositional engineering.^13,14^ A widely used method entails mixing cesium (Cs) and FA cations on the A-site of the perovskite crystal structure,^15^ thus favoring the desired perovskite α-phase formation.^16^ This is consistent with the Goldschmidt factor (*t* = 0.95) for the Cs_*x*_FA_1–*x*_PbI_3_ crystal structure with *x* = 0.15.^17^ In addition, a recent study on the nanostructure–property relationship of Cs_*x*_FA_1–*x*_PbI_3_ films reveals the crucial role of fine-tuning the Cs content to reduce the defect density to enhance electronic conductivity, device performance, and stability.^18^ Owing to these advantages, the fabrication of large-area Cs_*x*_FA_1–*x*_PbI_3_ devices has been attempted by various methods, such as chemical vapor deposition,^19^ blade/slot-die coating,^20,21^ screen-printing, and inkjet and spray coating.^22^ Notably, blade and slot-die coating have shown promising prospects for the future mass production of perovskite solar cells,^23−25^ despite the lower solubility of cesium halides and the more complex kinetics of nucleation and crystallization in comparison with MAPbI_3._^26^

For example, Bu et al. introduced methylammonium chloride (MACl) as an additive and the cosolvent *N*-methyl-2-pyrrolidone (NMP) in a Cs_0.12_FA_0.88_PbI_3_ ink to control the formation of the intermediate phase, thereby reaching a device efficiency of 15.3% for an aperture area of 205 cm^2^. Moreover, 22.4 cm^2^ unencapsulated devices [initial power conversion efficiency (PCE) = 19.2%] retained 80% of their efficiency after 1000 h at maximum power point (MPP) tracking under one sun illumination.^27^ Another method to control the perovskite film crystallization and morphology is to use other lead sources, such as lead acetate (Pb(OAc)_2_) or lead chloride (PbCl_2_).^28^ By blade-coating a lead acetate-based ink, Zhao et al. demonstrated that encapsulated Cs_0.17_FA_0.83_PbI_3_ mini-modules display a maximum efficiency of 18.8% on an aperture area of 10 cm^2^ with robust thermal stability along with negligible efficiency loss after 3300 h at 65 °C and a *T*_80_ of 327 h for nonencapsulated devices at MPP tracking under continuous LED light illumination (100 mW/cm^2^).^29^ Finally, Deng et al. demonstrated encapsulated blade-coated Cs_0.08_FA_0.92_PbI_3_ mini-modules reaching a certified efficiency of 18.6% on an aperture area of 30 cm^2^ and maintaining more than 90% of the initial efficiency after continuous operation for over 1000 h under one sun illumination near MPP tracking.^30^

However, the mechanisms of large-scale perovskite film formation are much less well-studied. In particular, it is crucial to be able to integrate *in situ* measurement tools to assess the nucleation and growth kinetics of these films. This will enable them to be linked to process parameters and perovskite formulations (e.g., the role of additives) and ultimately to the properties of the films themselves. In this work, we fabricate large-area FA-rich perovskite films by blade-coating a perovskite precursor solution of nominal composition Cs_0.15_FA_0.85_PbI_3_ in dimethylformamide (DMF) with N,N′-dimethylpropyleneurea (DMPU) and thiourea (TU) as additives. This specific perovskite composition was chosen by virtue of the already demonstrated performance and stability of spin-coated films.^15,18^ The ink formulation was adapted and optimized for the blade coating process to obtain compact films with micrometric-sized grains using two Lewis-base additives (DMPU and TU) instead of the commonly used dimethyl sulfoxide (DMSO). These two additive molecules have been studied over the past few years for spin coating^31,32^ and blade-coating processes,^25^ as they can modify the perovskite formation kinetics and thus the final film morphologies. The structures of the obtained films (reference film with additive DMPU and target film with both additives, DMPU and TU) were then studied by X-ray diffraction (XRD) measurements, transmission electron microscopy (TEM), and scanning electron microscopy (SEM). The blade-coated Cs_0.15_FA_0.85_PbI_3_ target film demonstrated a stacking fault-free α-cubic FAPbI_3_ perovskite structure, similar to films prepared by spin-coating. Using Fourier transform infrared (FTIR) and *in situ* absorption spectroscopy, the crystallization mechanism and specific role of thiourea were investigated. The results highlighted a strong interaction between TU and perovskite precursors in solution and a lower formation energy of the α-FAPbI_3_ phase in the presence of this additive. Finally, this optimized composition was used in 1 cm^2^ perovskite solar cells (PSCs) blade-coated on 5 × 5 cm^2^ substrates, with the best devices reaching a PCE of 16.9%.

## Results and Discussion

### Fabrication of Spin- and Blade-coated CsFA Perovskite Films

**Figure 1:**
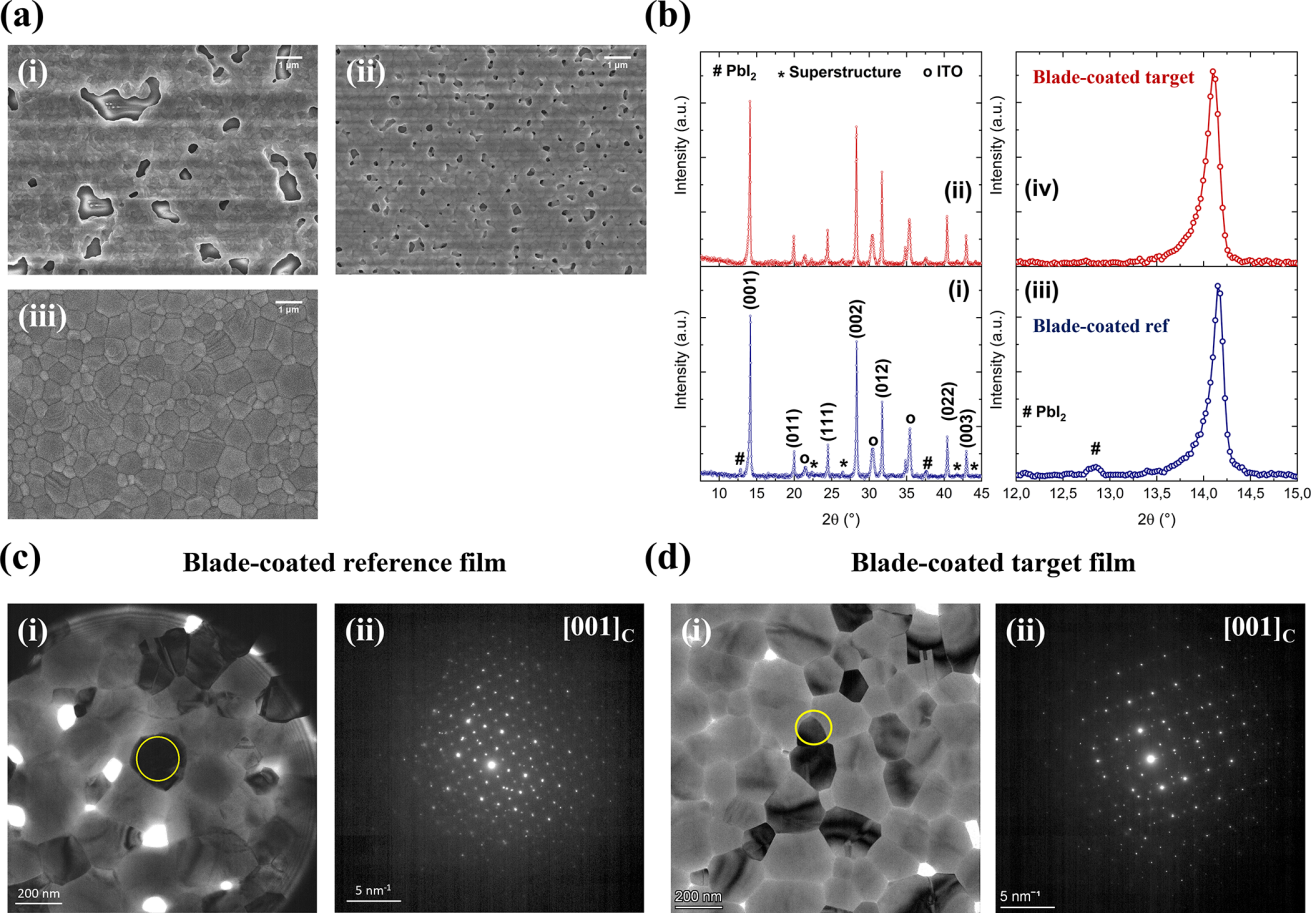
(a) Top-view SEM images of Cs_0.15_FA_0.85_PbI_3_ perovskite films (i) in DMF/DMSO, blade-coated, (ii) in DMF/DMPU, blade-coated (reference), and (iii) with 5%mol thiourea in DMF/DMPU, blade-coated (target) (scale bar = 1 μm). (b) XRD diffractograms of Cs_0.15_FA_0.85_PbI_3_ blade-coated thin films (i, iii) in DMF/DMPU (reference) and (ii, iv) with 5% mol thiourea in DMF/DMPU (target). (c, d) (i) BF TEM micrographs for Cs_0.15_FA_0.85_PbI_3_ perovskite blade-coated thin films (reference, target), indexed to a cubic FAPbI_3_ phase-oriented near the [001]_C_ zone axis. (ii) Associated SAED patterns (yellow circles indicate the aperture position for SAED pattern acquisition).

To characterize the additive-free perovskite, films produced from an ink of nominal composition Cs_0.15_FA_0.85_PbI_3_ (in DMF/DMSO 4:1 v/v) were deposited on glass/ITO substrates by both spin-coating and blade-coating. The spin-coated film exhibits a compact structure with relatively small-size domains (250 nm) (Figure S2a), while the blade-coated counterpart shows numerous micrometric-sized pinholes with smaller-sized perovskite domains (Figure 1ai) and coating nonuniformities (Figure S3 left). Those differences in film morphology can be explained by the different kinetics of nucleation and crystallization induced by the spin-coating (antisolvent step) and blade-coating processes (gas quenching).^33,34^ To obtain a compact morphology by blade-coating, we chose DMPU as a cosolvent, which we expect to inhibit nucleation and eventually modify crystallization kinetics on account of its strong donating character [O-donor with Gutmann’s Donor number (DN) = 34 kcal/mol^35^]. Consequently, an ink of nominal composition Cs_0.15_FA_0.85_PbI_3_ in DMF and DMPU (93:7 v/v) was prepared and the blade-coated film (reference film) displayed a mirror-like aspect, suggesting improved film homogeneity (Figure S3 right). However, pinholes of relatively small size could still be observed (Figure 1aii).

To overcome the presence of pinholes, thiourea was selected as a second additive, due to its Lewis basicity (S-donor that can form an adduct with Pb species, DN = 32 kcal/mol^36^) and to its -NH_2_ functional groups (possible hydrogen bonds formation with perovskite precursors).^37,38^ The corresponding top-view SEM images are displayed in Figure 1aiii showing dense pinhole-free micrometer-sized grains of blade-coated films (target film).

### Structural Characterizations of Blade-Coated Thin Films

The crystallographic structures of the perovskite films (reference and target), examined using XRD, are shown in Figure 1b. The diffraction peaks are assigned to the (001), (011), (111), (002), (012), (022), and (003) crystallographic planes of the α-cubic FAPbI_3_ perovskite structure in both samples (Figure 1bi,ii). Minor peaks corresponding to PbI_2_ and ITO are present, as well as peaks attributed to the cubic supercell structure.^39^ Moreover, we observe a peak shift toward higher angles (for example, ∼28.3° instead of ∼28.15°) for the (002) peak in comparison with pure FAPbI_3_, indicating lattice contraction and successful incorporation of Cs^+^ cations inside the perovskite structure.^18^ Furthermore, the intensity ratio between the (001) perovskite peak (14.1°) and the PbI_2_ peak (12.8°) suggests enhanced conversion to a perovskite phase for the target film, as no other secondary phase is detected (Figure 1biii,iv).

Inks of nominal composition Cs_0.15_FA_0.85_PbI_3_ (reference and target formulations) were solution-processed on ultrathin carbon-coated copper TEM grids by blade-coating. All process parameters were kept identical to previous experiments except for the blade height (decreased from 100 to 25 μm) to obtain an electron-transparent ∼150 nm-thin film. The TEM bright-field (BF) images and the corresponding selected-area electron diffraction (SAED) patterns for such perovskite thin films are presented in Figures 1c,d and S4. Note that the change of surface wettability for different substrates and/or process conditions can modify the morphology of the films to some extent, in comparison with those obtained on glass/ITO, as observed in references.^39,40^ The films deposited on TEM grids show relatively smaller domains compared to the films deposited on substrates examined by SEM. However, we assume here that the films’ crystallographic properties are representative of those of films used in devices.

For both compositions (blade-coated reference and target), the SAED patterns of a selection of grains could be indexed with a cubic FAPbI_3_ phase (*Pm**m* crystal structure) oriented near the [001]_C_ zone axis (Figure 1c,d) and the [011]_C_ zone axis (Figure S4). Crystallographic defects such as stacking faults or twinned domains were not observed in bright field images, which is coherent with TEM observations of spin-coated samples.^18^ Such defects could have a detrimental effect on the intrinsic stability of perovskite films. In addition, forbidden reflections in the cubic *Pm**m* phase were detected for both films (reference and target) (Figure S4) and could only be indexed to a cubic superstructure within the *Im* space group (already observed by XRD), attributed to octahedral tilting upon Cs-incorporation into the perovskite lattice.^39,41^ Overall, these results indicate that the optimized Cs-doped ink yields perovskite films with a similar microstructure regardless of the deposition process (spin-coating versus blade-coating). The presence of thiourea as an additive does not seem to impact the local structure, as could be the case with other additives (for example, MACl^39^).

### Interactions between Thiourea and Perovskite Precursors Probed by FTIR

**Figure 2:**
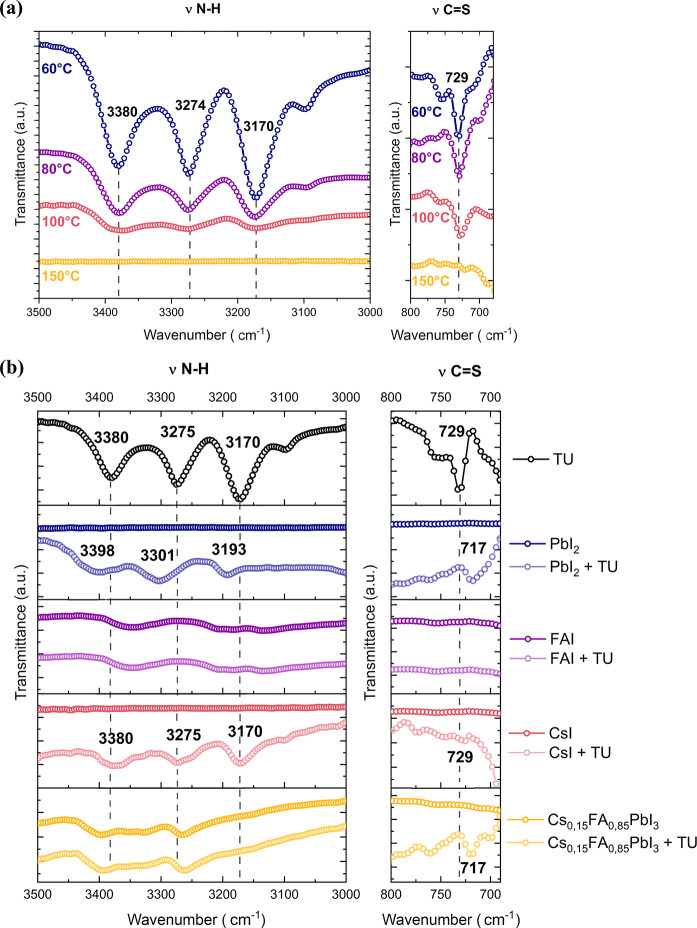
(a) FTIR spectra of thiourea after annealing at different temperatures (left) N–H stretching (right) C=S stretching. (b) FTIR spectra (taken at 80 °C) of thiourea (black curve), PbI_2_ and PbI_2_-thiourea (dark and bright blue), FAI and FAI-thiourea (dark and bright purple), CsI and CsI-thiourea (dark and bright pink), and PbI_2_–FAI-CsI and PbI_2_–FAI-CsI-thiourea (dark and bright yellow) (left) N–H stretching (right) C=S stretching.

Fourier transform infrared spectroscopy (FTIR) was used to evaluate the interactions between thiourea and the main components of the perovskite ink. Notably, the measurements were performed on semidry thin films deposited on crystalline silicon substrates and annealed at the indicated temperature (60, 80, 100, and 150 °C) for 2–3 min to remove the majority of the solvent while keeping the perovskite precursors/additives to some extent. The FTIR spectra of pure thiourea are presented in Figure 2a, showing the C=S stretching vibration appearing at 729 cm^–1^ and the N–H stretching modes at 3170, 3274, and 3380 cm^–1^. Upon annealing (from 60 to 150 °C), the intensity of the bands decreases with increasing temperature until no signal is detected at 150 °C, indicating that most of the thiourea is eliminated after the annealing step. However, thiourea could remain in low concentrations within films at grain boundaries and potentially improve the perovskite stability through a Pb–S binding.^42^

Figure 2b shows the FTIR spectra of pure thiourea as well as the different compounds of the ink (alone or mixed with thiourea): PbI_2_, PbI_2_-thiourea, FAI, FAI-thiourea, CsI, CsI-thiourea, and a mix of 0.15CsI-0.85FAI-PbI_2_ (also noted Cs_0.15_FA_0.85_PbI_3_) and Cs_0.15_FA_0.85_PbI_3_-thiourea acquired at 80 °C (see Materials and Experimental Methods section).

The C=S stretching vibration of thiourea appearing at 729 cm^–1^ is shifted to 717 cm^–1^ when it is mixed with PbI_2_. One can attribute this frequency shift to an interaction between lone pair electrons on S and the 5d empty orbital of Pb, characterized by electron cloud migration from C=S to Pb and the decrease of the force constant of the C=S bond.^32^ A similar shift is observed when thiourea and the perovskite ink of nominal composition Cs_0.15_FA_0.85_PbI_3_ are mixed, indicating a similar interaction. On the contrary, such a shift in C=S stretching vibrations is not observed when mixed with CsI, suggesting that thiourea and CsI do not interact.

The N–H bands of thiourea shift to 3193, 3301, and 3402 cm^–1^ when mixed with PbI_2_. Those N–H bond shift changes can be due to either a direct interaction of the N–H bond with PbI_2_ through hydrogen bonds, or a possible change in C=S bonds through an interaction with PbI_2_. Similarly, as above, no shift in thiourea’s N–H vibration is observed when mixed with CsI, corroborating the noninteraction between those two components. For the mixture with FAI and the perovskite ink, the presence of wide vibrational bands around 3100–3500 cm^–1^ (corresponding to the N–H stretching mode of FAI) does not allow for the detection of any signal from the thiourea molecule. However, we observe a shift of the N–H bands toward higher wavenumbers (when mixing FAI with PbI_2_ and CsI), indicating a possible hydrogen bonding between FA^+^ ions and iodides in [PbI_6_]^4−^.^43^ One can also note the additional interaction between DMPU and the perovskite precursors, characterized by the shift of the C=O band of DMPU (from 1633 to 1613 cm^–1^) as well as by the presence of the peak of the adduct FAI–DMF–DMPU at 1716 cm^–1^ (corresponding to the C=N stretch vibration of the FAI molecule, observed at 1697 cm^–1^ when alone^25^) (Figure S5). From FTIR data, thiourea interacts with the perovskite precursors (PbI_2_) and this generation of intermediate additive-PbI_2_ complexes could hence impact the overall perovskite formation kinetics.

### Kinetics of Perovskite Formation

To gain some insight into the different kinetic processes and to understand the specific role of thiourea, *in situ* real-time absorption spectra of spin-coated and blade-coated perovskite films were recorded during the annealing step at different temperatures (80, 100, 125, and 150 °C). By plotting the average absorption on the range [600–800] nm, where the incident light is strongly absorbed by perovskites but negligible for the intermediate species, the amount of perovskite can be evaluated as it forms. As the perovskite formation process is thermally driven in this specific configuration (blade-coating then annealing step), it becomes faster as the temperature increases, as shown by the recorded spectra (Figures S8–S11).

This behavior can be further analyzed by applying Moore’s methodology^28^ and Mittemeijer’s formula for isothermal kinetic data for solid-state transformations (without considering the type of nucleation or growth^44^), which is described by the following equation:
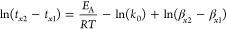
1where *t*_*xn*_ is the time at which the transformed fraction is *x*_*n*_, β_*xn*_ is a state property related to the transformed fraction of the film (quantified here by the film absorption spectra), *E*_A_ is the effective activation energy, *R* is the gas constant, *T* is the temperature, and *k*_0_ is the crystallization rate constant prefactor.

**Figure 3:**
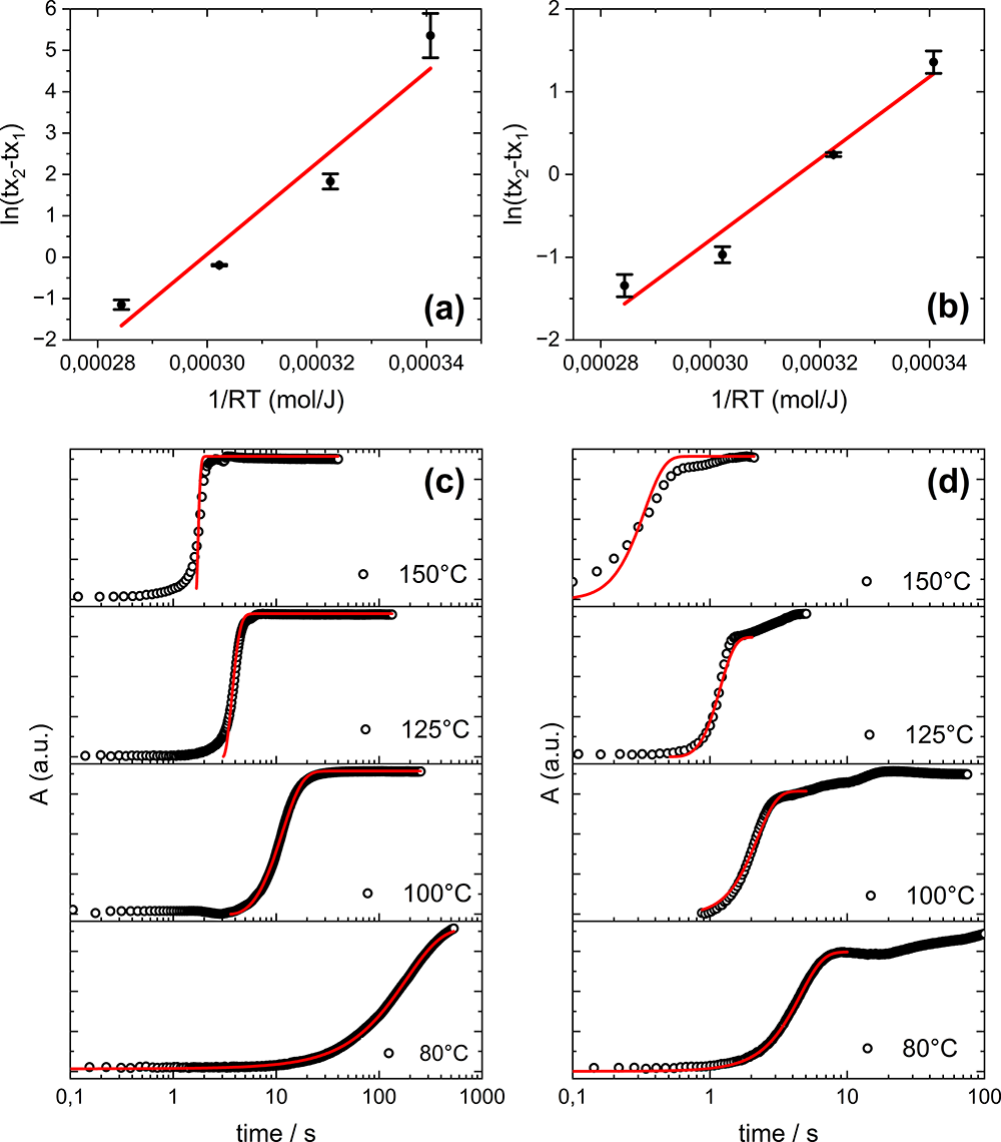
: Plots constructed from eq 1 to extract the activation energy *E*_A_ for (a) blade-coated reference, the slope of the line is 114 ± 21 kJ/mol, and (b) blade-coated target, the slope of the line is 49 ± 6 kJ/mol. Absorbance (average values on the [600–800] nm range) derived from *in situ* optical measurements as a function of annealing time for (c) reference and (d) target blade-coated Cs_0.15_FA_0.85_PbI_3_ films annealed at different temperatures (black circles: experimental points; red lines: fits with JMA kinetic model).

By plotting ln(*t_x_*_2_–*t_x_*_1_) versus 1/*RT* and extracting the slope of the linear fit, we can evaluate the activation energy for the whole process (nucleation and crystal growth are not decoupled). The experimental points and linear fits are depicted in Figure 3a,b and Table S1 with starting and ending values of *x*_1_ = 0.3 and *x*_2_ = 0.8 for both reference and target films, respectively. Such values for *x*_1_ and *x*_2_ are chosen to minimize error as absorption values for *x* below *x*_1_ or above *x*_2_ are quite sensitive to error propagation because of small values for d*A*/d*t*.

The slope from the linear fit yields an effective activation energy of 114 ± 21 kJ/mol for the reference film and 49 ± 6 kJ/mol for the target film. Hence, it is found that the activation energy for perovskite formation is divided by two when adding thiourea to the precursor ink, which is similar to recent results obtained on spin-coated MAPbI_3_ films with thiourea and urea as additives.^45,46^

As an indication, considering the difficulty in comparing two different coating processes from a kinetic point of view, the activation energy for the spin-coating process is calculated at 38 ± 4 kJ/mol for the reference ink formulation and at 27 ± 3 kJ/mol for the target ink formulation (Figures S12–S17). This decrease in activation energy upon addition of thiourea is also observed when films are spin-coated, but to a lesser extent, probably due to the antisolvent step which has been proven to increase the nucleus density during film formation.^47^ Note the strong increase in absorption signal around 550 nm for all spin-coated films suggesting a secondary nucleation as recently observed.^48^ Then the bulk perovskite phase starts to form, indicated by the increasing optical absorption toward the desired band edge (800 nm). Note also that the obtained activation energy values are consistent with those previously reported for FAPbI_3._^49,50^ The formation kinetics and activation energy values obtained can be coherently related to the spin- and blade-coated films morphologies shown in Figures 1, S2 and S3.

The kinetics analysis can then be completed by modeling the absorbance data with the Johnson–Mehl–Avrami (JMA) model^51^ described by the following equations:
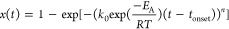
2
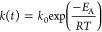
3where *k*_0_ is the crystallization rate constant prefactor, *k* is the crystallization rate constant, *E*_A_ is the effective activation energy, *R* is the gas constant, *T* is the temperature, *t*_onset_ is a delay time introduced to consider the heat transport effect, and *n* is the growth exponent, which describes the dimensionality of the growth and should be between 1 and 4.^52^

Experimental points and the resulting JMA models are presented in Figure 3c,d for both reference and target films. The detailed fitting parameters are presented in Table S2. As expected from the activation energy estimation and from the experimental and fitting parameters (Tables S1, S2, and Figure S18), the reference film displays slower crystallization kinetics in comparison with the target one. The Avrami parameter for the target film (*n* ∼ 2.8) suggests a 2D layer growth of the perovskite film, whereas the reference film is more prone to growth on one dimension only (*n* ∼ 1.8) while the nucleation rate is kept constant.^53^

Hence, those kinetic data demonstrate the faster crystallization of the TU-treated target films along with reduced activation energy, which contributes to the obtained optimal structure of compact pinhole-free blade-coated films. Those kinetic changes are suggested to result from the strong interaction between the Lewis base (TU) and the precursor compounds (PbI_2_). Similar results were obtained lately on spin-coated MAPbI_3_ films^45^ with the generation of intermediate TU additive-MAI-PbI_2_ complexes that can effectively reduce the energy barrier and regulate rapid nucleation and crystal growth at lower annealing temperatures. This conversion process could be then further visualized by *in situ* optical microscopy observations on top of coated inks^54^ and SEM observations of perovskite films at different annealing times/temperatures.^55^ It might also be interesting to complement this study with phase field simulations to link nucleation rate, growth rate, and final film morphology and properties (e.g., with various amounts of TU) as studied in previous studies^56,57^ to obtain a composition optimized both for the deposition process (faster kinetics in the case of blade-coating) and for the final film quality (high crystallinity and defect-free perovskite).

Another interesting piece of information provided by the crystallization kinetics data is the presence of two distinct crystal growth stages for all target films (spin- and blade-coated) with a second slowed-down mechanism, as depicted in Figures 3d and S13. Hence, experimental data and JMA model are diverging at high conversion rates when small grains grow into larger ones. This divergence could be explained by a phenomenon of Ostwald ripening, either limited by the mass transport (diffusion-limited kinetic regime) or by attachment of small grains on the larger ones (interface-reaction kinetic regime).^58,59^

### Blade-Coated Perovskite Devices

**Figure 4:**
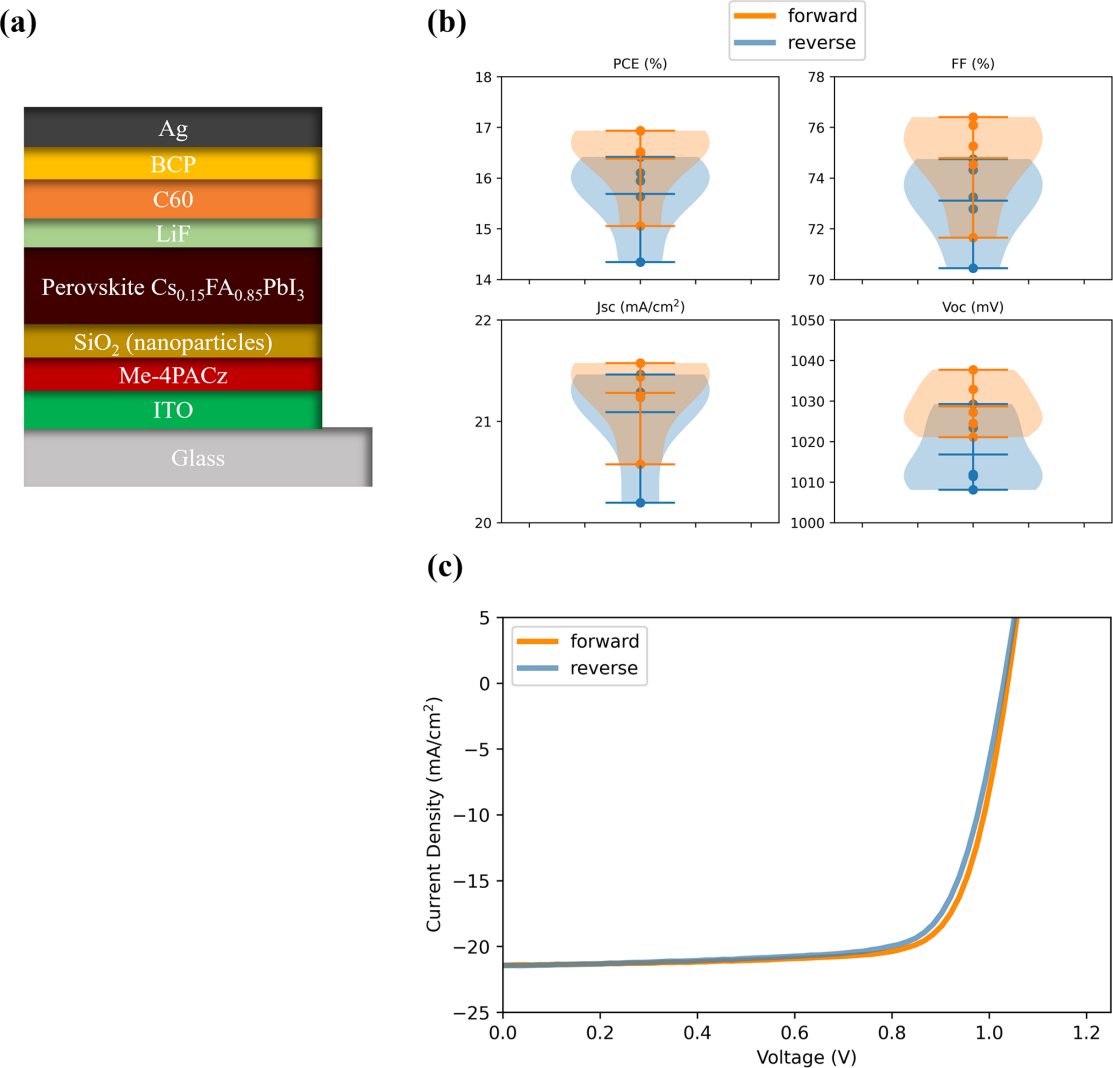
: (a) Scheme of the blade-coated perovskite solar cell, (b, c) device characterization of blade-coated Cs_0.15_FA_0.85_PbI_3_ perovskite solar cells (b) photovoltaic parameters: PCE, FF, *J*_sc_, and *V*_oc_ of blade-coated solar cells. (c) *J*–*V* characteristics of champion blade-coated target Cs_0.15_FA_0.85_PbI_3_ device (mask area: 1 cm^2^).

Finally, the target composition is implemented within blade-coated perovskite solar cells with the device structure described in Figure 4a (see also the Materials and Experimental Methods section). The photovoltaic characteristics of such devices (1 cm^2^) are summarized and plotted in Figure 4b, and the typical *J*–*V* curves of the champion device are shown in Figure 4c. The PCE of the champion device is 16.9% with an open-circuit voltage (*V*_oc_) of 1038 mV, a short-circuit current density (*J*_sc_) of 21.58 mA/cm^2^, and a fill factor (FF) of 76.4%. As shown, blade-coated devices show a maximum efficiency of close to 17%. We note that the attained efficiency is 2% lower than the reported maximum efficiency of spin-coated devices for the same composition tested on 0.1 cm^2^^18^ and further optimization regarding perovskite thickness, stack optimization, and extra passivation layer is needed to bridge the gap.

## Conclusions

In summary, this work has been dedicated to the transfer of the optimized Cs_0.15_FA_0.85_PbI_3_ film deposition from a lab-scale process to a more industrially compatible coating method, i.e., blade-coating. By combining two Lewis bases [N,N′-dimethylpropyleneurea (DMPU) and thiourea (TU)], compact high-quality perovskite films with micronic-size domains were obtained by blade-coating. Structural characterizations of such films at both macro- and nanoscale show that the chosen composition was easily transferable from small-area processes to larger areas films, with high crystal quality perovskite films obtained by blade-coating. The role of thiourea as an additive and kinetic controller is elucidated by FTIR spectroscopy and *in situ* absorption during the process. The formation of PbI_2_-thiourea adducts lowers the formation energy of α-FAPbI_3_, helping to form high-quality perovskite films on a larger scale. This optimized composition was then tested for solar cell fabrication with a maximum PCE of 16.9% for a surface area of 1 cm^2^. Thus, these results provide a promising pathway to obtain high-quality FA-rich perovskite films by scalable and industrial processes.

## Materials and Experimental Methods

Inks, films, and device fabrication processes are done inside a glovebox filled with a N_2_ atmosphere.

### Materials

Anhydrous ethanol (EtOH), anhydrous dimethylformamide (DMF), anhydrous dimethyl sulfoxide (DMSO), anhydrous N,N′-dimethylpropyleneurea (DMPU), and thiourea (TU) were purchased from Sigma-Aldrich. Formamidinium iodide (FAI) was purchased from Dyenamo. Lead iodide (PbI_2_), cesium iodide (CsI), and (4-(3,6-dimethyl-9H-carbazol-9-yl)butyl)phosphonic acid (Me-4PACz) were purchased from TCI. All chemicals were used as received.

### Perovskite Ink Preparation

The nominal perovskite composition used is Cs_0.15_FA_0.85_PbI_3_. The perovskite inks are prepared by dissolving 461.0 mg of PbI_2_, 146.2 mg of FAI, and 38.9 mg of CsI in 1 mL of solvent, either DMF/DMSO (4:1 v/v) or pure DMF, to prepare 1 M Cs_0.15_FA_0.85_PbI_3_ solutions. For the DMPU-containing inks, 73 μL (0.56 mmol) of DMPU was added to 1 mL of solution. For the thiourea-containing inks, 3.8 mg (0.05 mmol, 5 mol % vs Pb) are added to 1 mL of solution.

### Perovskite Thin Film Preparation

The preparation of spin-coated films is described in ref (18).

The preparation of blade-coated films is described below: cleaned ITO/glass substrates (5 × 5 cm^2^ aperture area) are treated with UV-Ozone for 15 min just before coating. Then 50 μL of the perovskite precursor solution is dropped with a gap of 100 μm between the doctor blade and the substrate and is coated at a speed of 5 mm/s. The temperature of the coating bed is 35 °C. Right after the doctor blade, a gas knife (N_2_, 60 L/min) is used to remove the excess solvent during coating. The deposited films are then immediately annealed at 150 °C for 15 min.

### Perovskite Solar Cell Preparation

Cleaned ITO/glass substrates (5 × 5 cm^2^ aperture area) are treated with UV-Ozone for 15 min just before deposition. Then, thermal evaporation of approximately 4 nm of Me-4PACz is performed on the substrates in a homemade evaporation system (base pressure <2 × 10^–6^ mbar, working pressure >3 × 10^–6^ mbar, evaporation rate of 0.1 Å s^–1^ as measured by quartz crystal microbalance). The deposited films are then immediately annealed at 100 °C for 15 min. Then, 50 μL of colloidal suspension of SiO_2_ nanoparticles (0.2 wt % in EtOH, average size = 30 nm) is deposited with a gap of 100 μm between the doctor blade and the substrate and is coated at a speed of 30 mm/s, with a gas knife (N_2_, 60 L/min) at room temperature. The deposited films are then immediately annealed at 100 °C for 10 min. Then 50 μL of the perovskite precursor solution is deposited with a gap of 100 μm between the doctor blade and the substrate and is coated at a speed of 5 mm/s, with a gas knife (N_2_, 60 L/min) and a coating bed temperature of 35 °C. The deposited films are then immediately annealed at 150 °C for 15 min. Finally, the stack is completed with thermal evaporation of lithium fluoride (LiF) (1 nm, 0.1 Å/s), fullerene (C60) (20 nm, 0.2 Å/s), bathocuproine (BCP) (5 nm, 0.1 Å/s) on a full area, and silver (Ag) through a shadow mask on the samples (130 nm, 1.5 Å/s).

### For TEM Experiments

The perovskite solutions are directly blade-coated on ultrathin carbon-coated copper TEM grids that are stuck to the ITO/glass substrate using a Kapton tape. Note down here that the TEM specimens are exposed to only 5 min of UV-ozone treatment. The gap height is decreased to 25 μm to obtain ∼150 nm-thick films. To minimize any environmental potential-induced degradations such as moisture exposure, the samples are placed in a nitrogen (N_2_)-filled cylinder immediately after deposition inside the glovebox and then transferred into the TEM chamber in less than 5 min of air exposure.

BF micrographs and SAED patterns are acquired on a Talos F200S TEM operated at 200 kV. To minimize any possible electron beam-induced artifacts, we used low-dose TEM imaging conditions, with an electron dose rate of ∼1 eÅ^–2^ s^–1^. All of the TEM BF micrographs and diffraction patterns are recorded from previously unexposed regions of the sample. Furthermore, the specimen is never tilted, and the SAED patterns are taken at the crystal orientation in which the domains are found. A small, selected area aperture of 10 μm in size is used to enable the record of localized DPs. The SAED patterns are analyzed using the JEMs software.^60^

### SEM

SEM images are acquired with an acceleration voltage of 5 kV and probe current of 28 μA on a JEOL 7500 TFE microscope.

### XRD

XRD is carried out in an Empyrean diffractometer (Panalytical) equipped with a PIXcel-1D detector. The XRD patterns are obtained using Cu Kα radiation (wavelength of 1.54 Å).

### FTIR

1 M reference solutions (PbI_2_, FAI, CsI, PbI_2_ with 85 mol % FAI, and 15 mol % CsI) and 1 M thiourea-containing solutions (TU, PbI_2_ with 5 mol % TU, FAI with 5 mol % TU, CsI with 5 mol % TU, PbI_2_ with 85 mol % FAI, 15 mol % CsI, and 5 mol % TU) are spin-coated on silicon wafers (3500 rpm during 35s) and then annealed at different temperatures (60, 80, 100, and 150 °C) during 2–3 min. The FTIR spectra are then recorded on a Bruker Vertex 80 spectrometer.

### *In Situ* Optical Measurements

*In situ* optical measurements are performed in reflection mode with a bifurcated fiber (Thorlabs), where one arm leads to the spectrometer (OceanInsight, Flame) and the other arm to a white light illumination source (tungsten halogen lamp from OceanOptics LS-1). The measurements were performed with an integration time of 10 ms per spectrum. The substrates are precoated with ∼150 nm Ag on the back of the substrate to maximize reflectivity without changing the top surface (structure: Ag/glass/perovskite). The spot size is ∼0.5 cm^2^, and the illumination intensity is 5–10 mW/cm^2^. The data processing is detailed in Note S1.

### Device Characterizations

In-house current–voltage (JV) measurements are obtained using a two-lamp (halogen and xenon) class AAA WACOM sun simulator with an AM1.5 G irradiance spectrum at 1000 W m^–2^. Shadow masks are used to define the illuminated area (here, 1 cm^2^). The cells are measured with a scan rate of 100 mV s^–1^ (using an integration time of 0.1 s and a delay of 0.1 s for each data point).

## Supporting Information Available

Solvents and additives used in this work; top-view SEM images of spin-coated Cs_0.15_FA_0.85_PbI_3_ perovskite films; blade-coated Cs_0.15_FA_0.85_PbI_3_ perovskite films; BF TEM micrographs for Cs_0.15_FA_0.85_PbI_3_ perovskite blade-coated thin films (reference, target) indexed to a cubic superstructure FAPbI_3_ phase-oriented near the [011]_C_ zone axis and near the [112]_C_ zone axis; FTIR spectra of PbI_2_–FAI-CsI dissolved in DMF/DMSO and in DMF/DMPU; description of the data analysis route for *in situ* optical measurements; heatmaps derived from *in situ* optical measurements for blade-coated Cs_0.15_FA_0.85_PbI_3_ films (reference) annealed at 80 °C; absorbance spectra at different times during the annealing step for blade-coated Cs_0.15_FA_0.85_PbI_3_ films (reference) annealed at 80 °C; heatmaps derived from *in situ* optical measurements for blade-coated and spin-coated Cs_0.15_FA_0.85_PbI_3_ films (reference, target) annealed at different temperatures; spectra derived from *in situ* optical measurements for blade-coated and spin-coated Cs_0.15_FA_0.85_PbI_3_ films (reference, target) annealed at different temperatures; plots constructed from eq 1 to extract the activation energy E_A_ for a spin-coated film Cs_0.15_FA_0.85_PbI_3_ films (without TU additive in DMF/DMSO); plots constructed from eq 1 to extract the activation energy E_A_ for a spin-coated film (with TU additive in DMF/DMSO); experimental parameters used for Mittemeijer’s formula and obtained linear fit parameters; kinetic parameters extracted from *in situ* optical measurements with the JMA kinetic model; and plots of ln(*k*) vs 1/*T* for blade-coated perovskite films (reference and target) (PDF)
