# Supplementary material for: Blade-Coating of High Crystallinity Cesium-Formamidinium Perovskite Formulations
Source: ACS Appl Mater Interfaces. 2024 Jul 1;16(28):36557–66. doi: 10.1021/acsami.4c04706 (PMC11261561; doi:10.1021/acsami.4c04706)
Supplement: Supplementary file 1 — am4c04706_si_001.pdf [file am4c04706_si_001.pdf]

Supporting Information

Blade-Coating of High Crystallinity Cesium-Formamidinium Perovskite Formulations

Authors: Anaël Jaffrès[1, 2], Mostafa Othman[1*], Felipe Saenz[2], Aïcha Hessler-Wyser[1], Quentin Jeangros[2], Christophe Ballif[1, 2], Christian M. Wolff[1*]

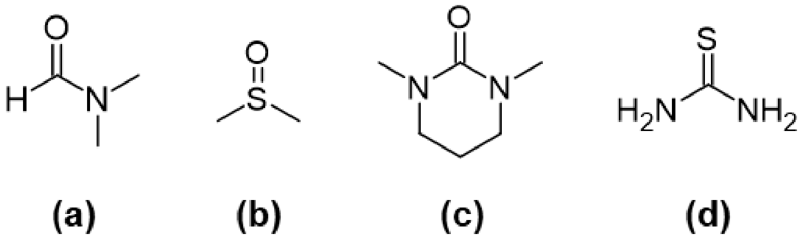

Figure S1 : Solvents and additives used in this work (a) DMF (b) DMSO (c) DMPU (d) TU.

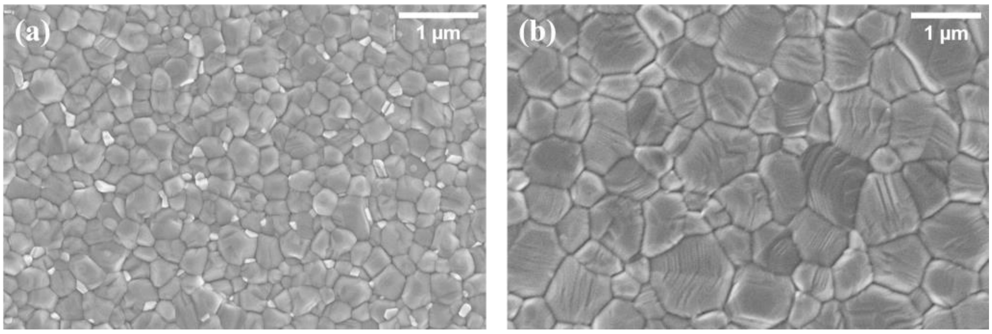

Figure S2 : Top-view SEM images of spin-coated $Cs_{0.15}FA_{0.85}PbI_3$ perovskite films (a) in DMF/DMSO, (b) with 5%mol thiourea in DMF/DMSO (scale bar = 1 µm).

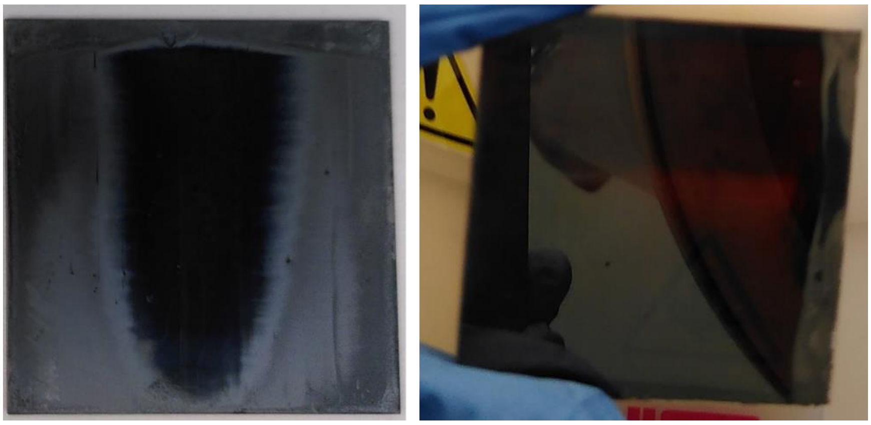

Figure S3 : Blade-coated $Cs_{0.15}FA_{0.85}PbI_3$ perovskite films (left) in DMF/DMSO (right) in DMF/DMPU (scale: 5x5 cm^2 samples).

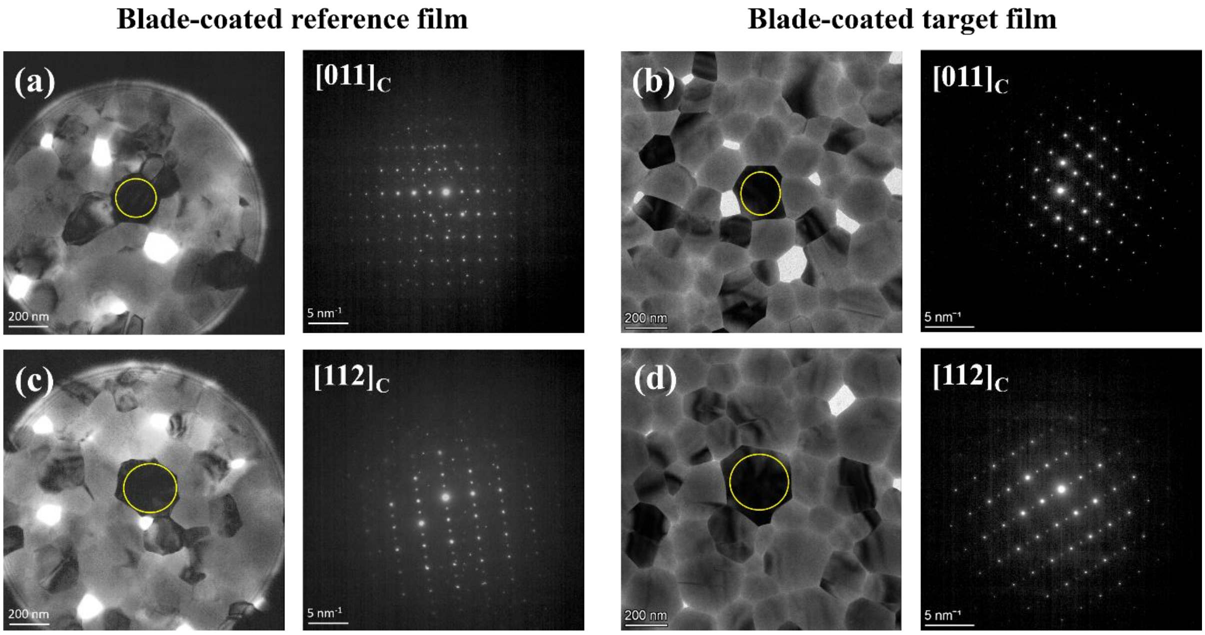

Figure S4 : (a, b) BF TEM micrographs for $Cs_{0.15}FA_{0.85}PbI_3$ perovskite blade-coated thin films (reference, target) indexed to a cubic superstructure $FAPbI_3$ phase oriented near $[011]_C$ zone axis and the associated selected-area electron diffraction patterns (yellow circles indicate the position of the selected-area for SAED pattern acquisition). (c, d) BF TEM micrographs for $Cs_{0.15}FA_{0.85}PbI_3$ perovskite blade-coated thin films (reference, target) indexed to a cubic superstructure $FAPbI_3$ phase oriented near $[112]_C$ zone axis and the associated selected-area electron diffraction patterns (yellow circles indicate the position of the selected-area for SAED pattern acquisition).

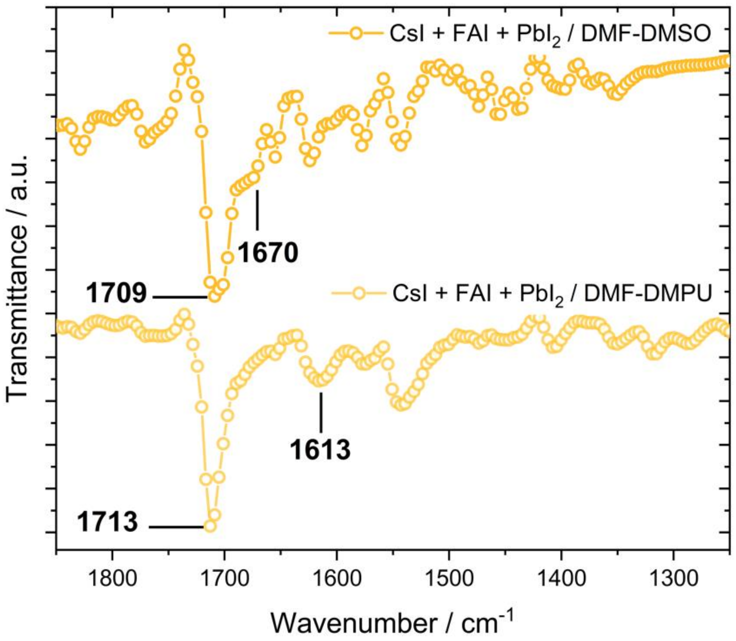

Figure S5 : FTIR spectra of PbI_2-FAI-CsI dissolved in DMF/DMSO (dark yellow) and in DMF/DMPU (bright yellow).

Note 1

The absorption was calculated according to the following equation[1,2,3]:

$$A(\lambda) = -\log_{10} \frac{(I(\lambda) - I_{dark}(\lambda))}{(I_{blank}(\lambda) - I_{dark}(\lambda))} \qquad (1)$$

where A(λ) represent the absorption at a specific wavelength, I(λ) represent the light intensity at a specific wavelength, $I_{blank}(\lambda)$ represent a bright reference spectrum recorded with the substrate beneath the probe and the light source turned on and $I_{dark}(\lambda)$ represent a dark reference spectrum with the light source turned off.

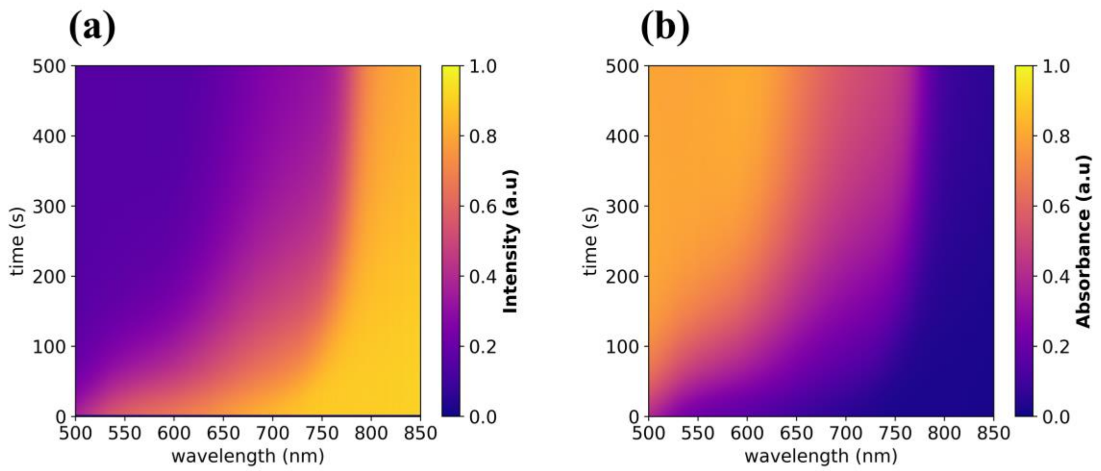

Figure S6: Heatmaps derived from *in situ* optical measurements for blade-coated $Cs_{0.15}FA_{0.85}PbI_3$ films (reference) annealed at 80°C (a) light intensity corrected with bright reference and dark reference (b) absorbance.

Figure S6 shows typical heatmaps obtained from in situ optical measurements. **Figure S7** shows typical absorption spectra at different times. For the kinetic analysis, the absorbance values are averaged between 600 and 800 nm and plotted versus time.

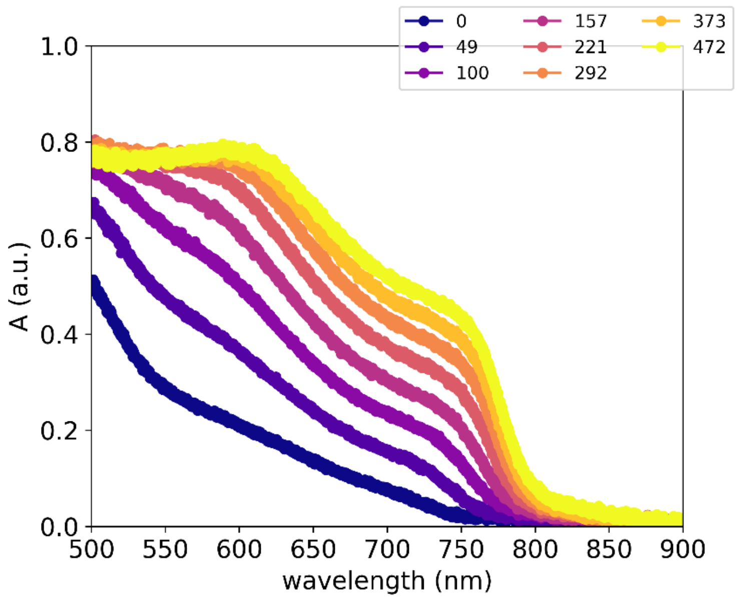

Figure S7: Absorbance spectra at different times during the annealing step for blade-coated $Cs_{0.15}FA_{0.85}PbI_3$ films (reference) annealed at 80°C. The legend indicates the values of time (in s).

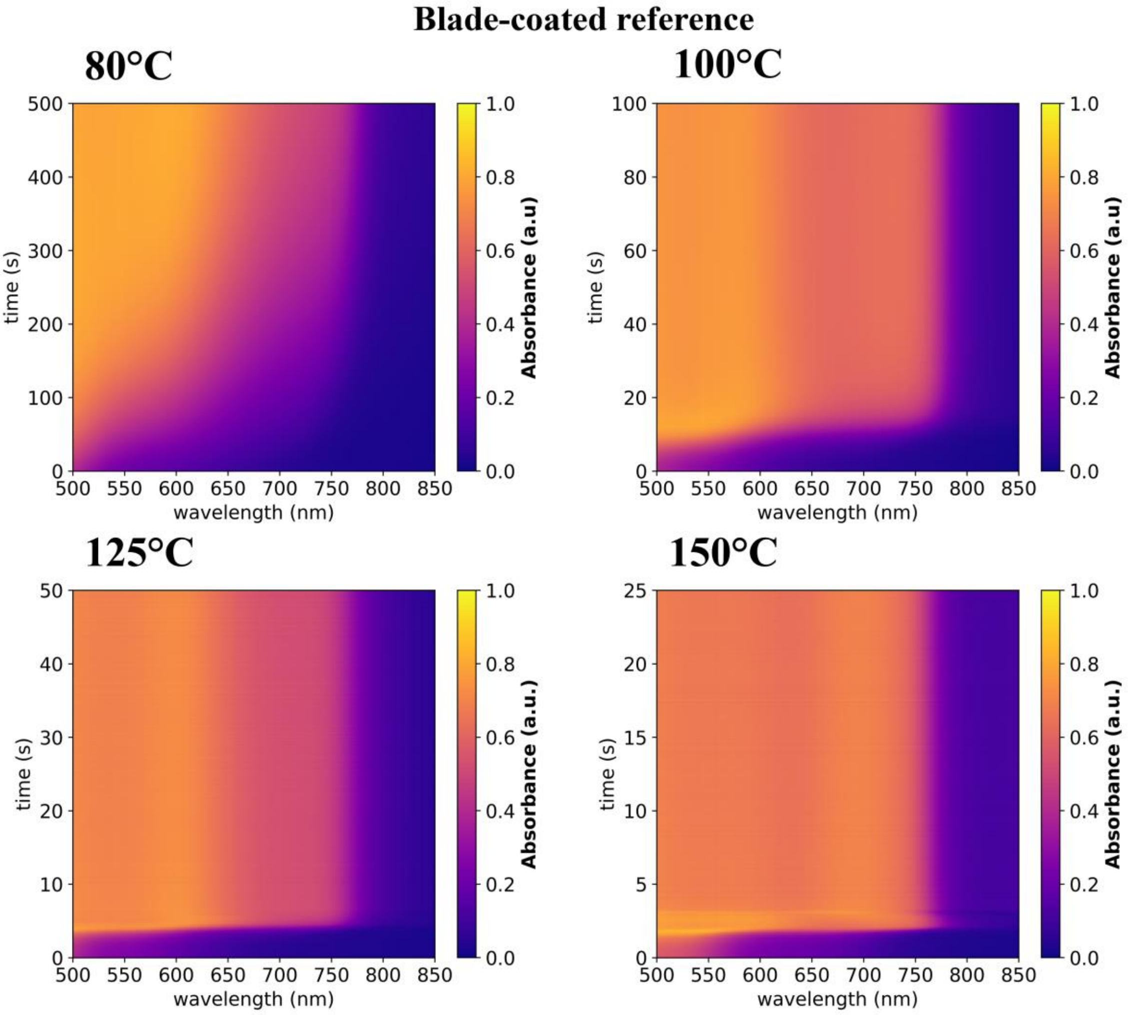

Figure S8 : Heatmaps derived from *in situ* optical measurements for blade-coated $Cs_{0.15}FA_{0.85}PbI_3$ films (reference) annealed at different temperatures.

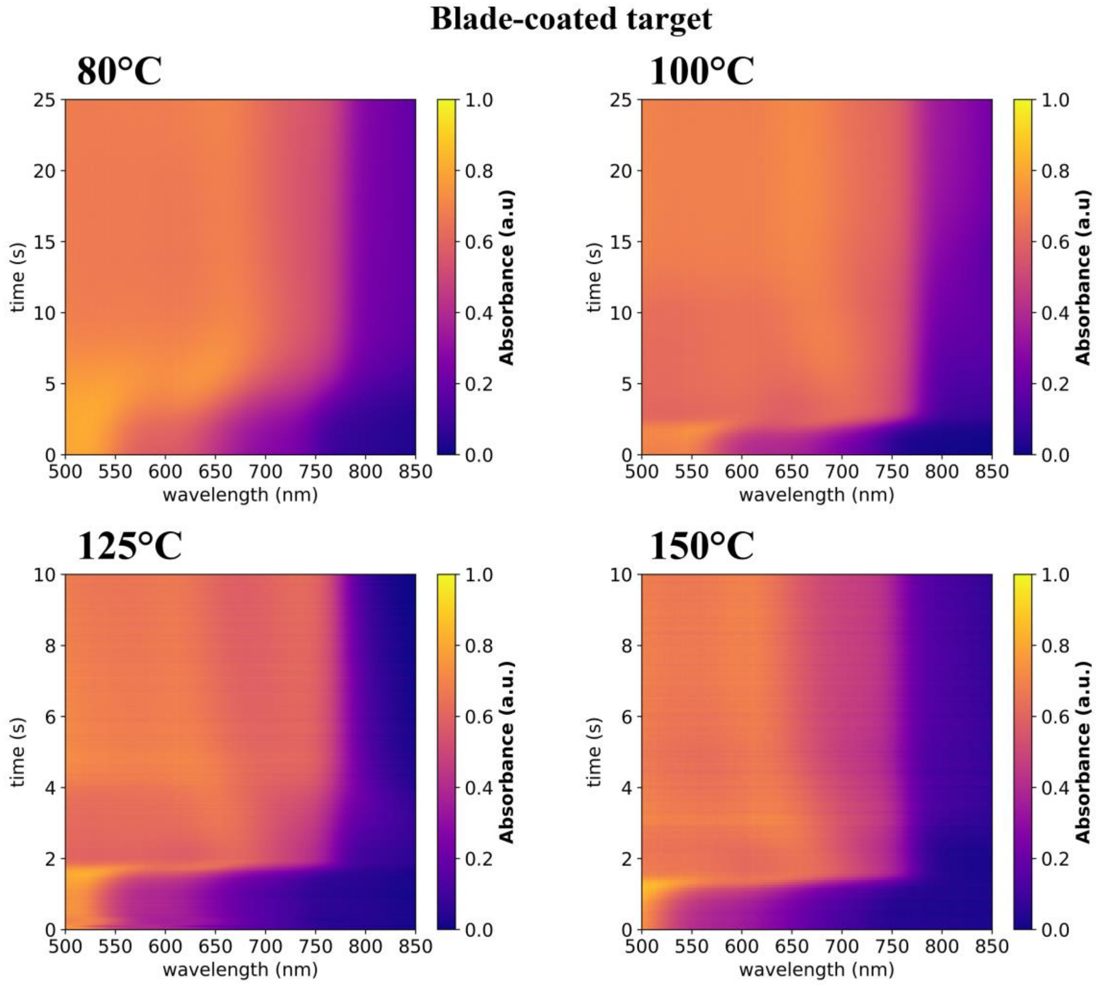

Figure S9 : Heatmaps derived from *in situ* optical measurements for blade-coated $Cs_{0.15}FA_{0.85}PbI_3$ films (target) annealed at different temperatures.

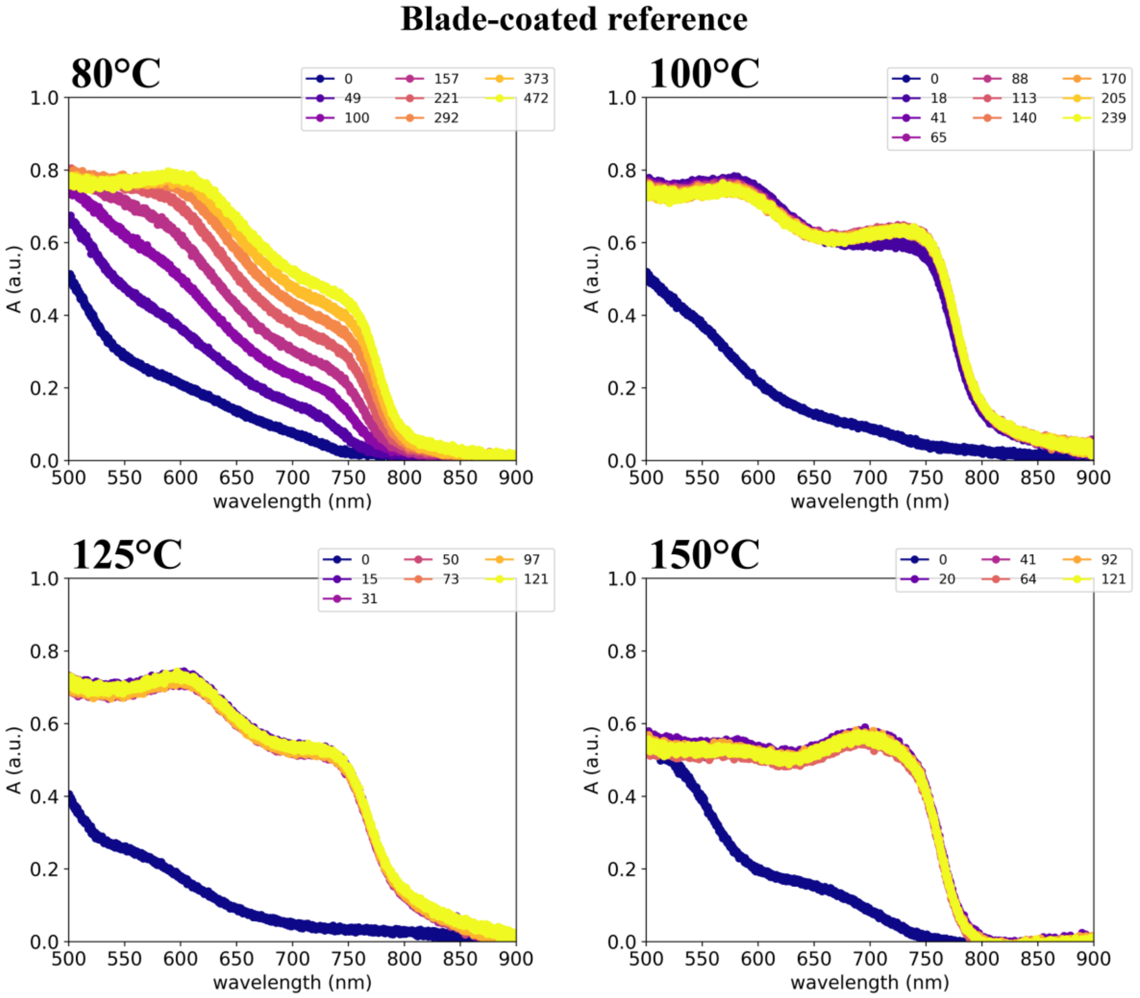

Figure S10 : Spectra derived from *in situ* optical measurements for blade-coated $Cs_{0.15}FA_{0.85}PbI_3$ films (reference) annealed at different temperatures. The legend indicates the values of time (in s).

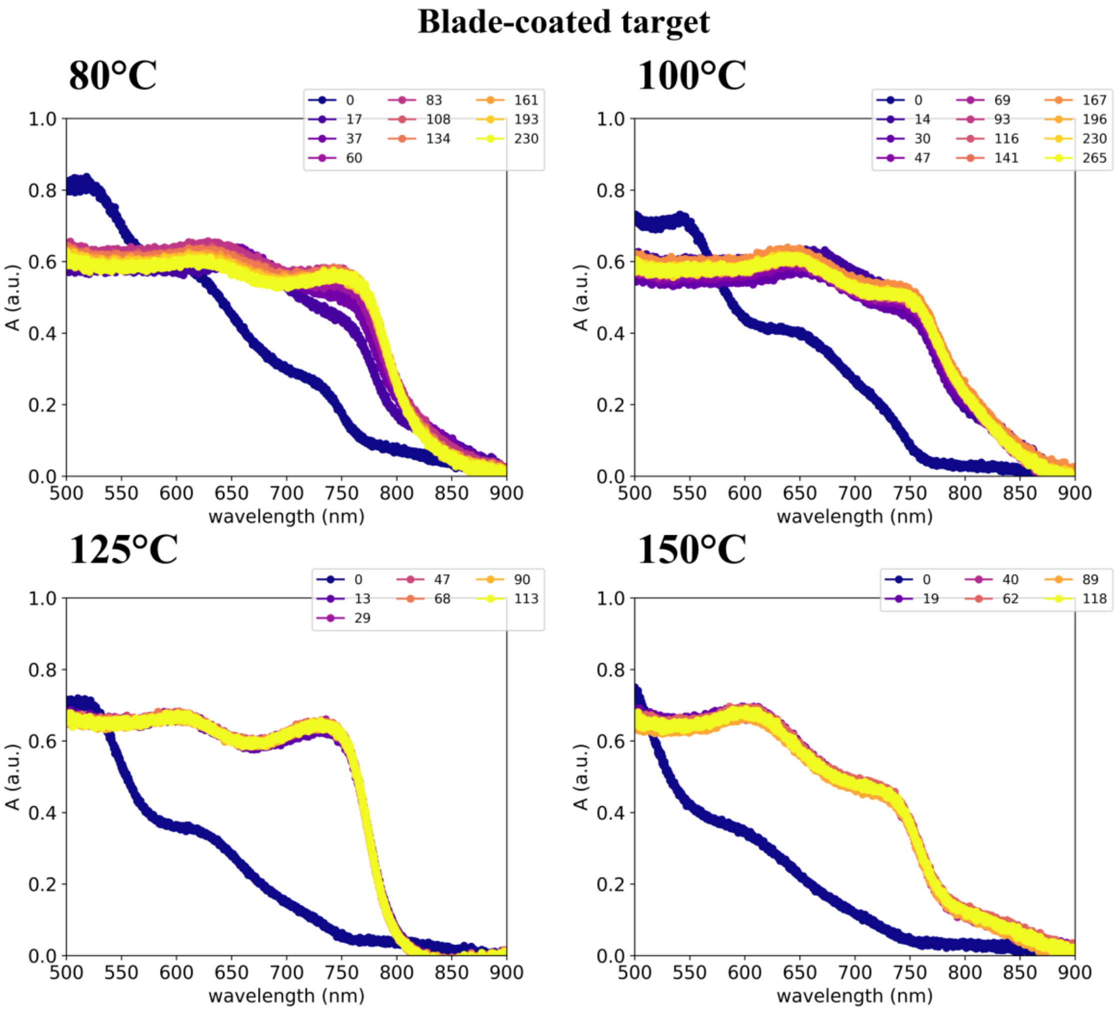

Figure S11 : Spectra derived from *in situ* optical measurements for blade-coated $Cs_{0.15}FA_{0.85}PbI_3$ films (target) annealed at different temperatures. The legend indicates the values of time (in s).

Spin-coated reference

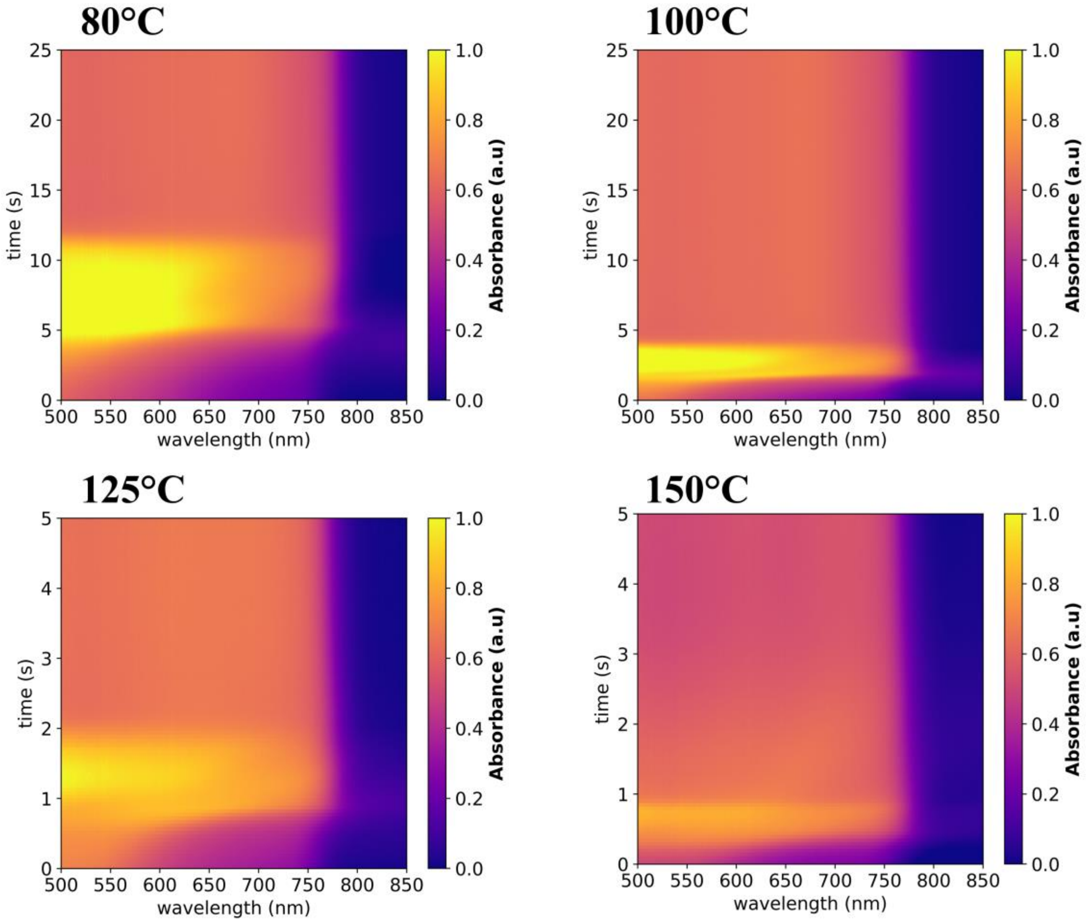

Figure S12 : Heatmaps derived from *in situ* optical measurements for spin-coated $Cs_{0.15}FA_{0.85}PbI_3$ films (reference) annealed at different temperatures.

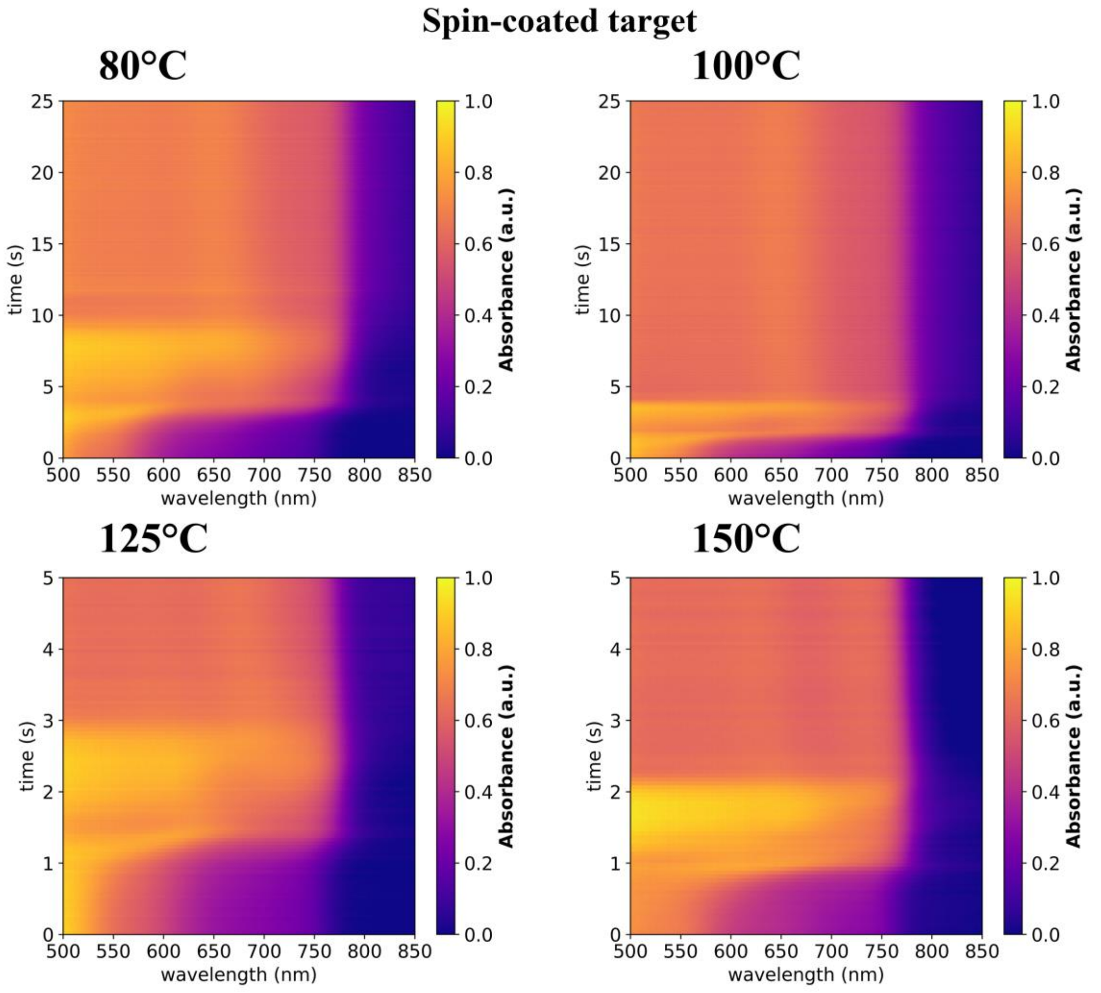

Figure S13 : Heatmaps derived from *in situ* optical measurements for spin-coated $Cs_{0.15}FA_{0.85}PbI_3$ films (target) annealed at different temperatures.

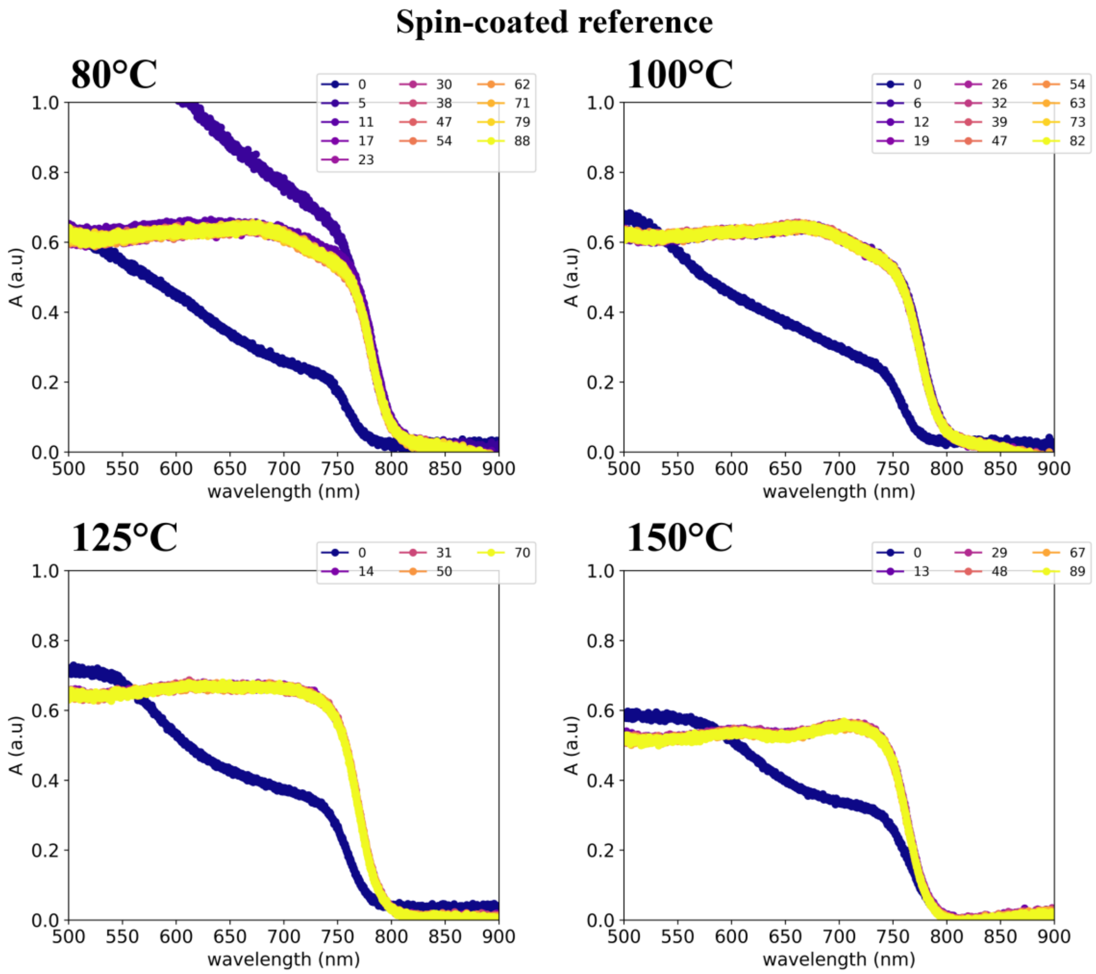

Figure S14 : Spectra derived from in situ optical measurements for spin-coated $Cs_{0.15}FA_{0.85}PbI_3$ films (reference) annealed at different temperatures. The legend indicates the values of time (in s).

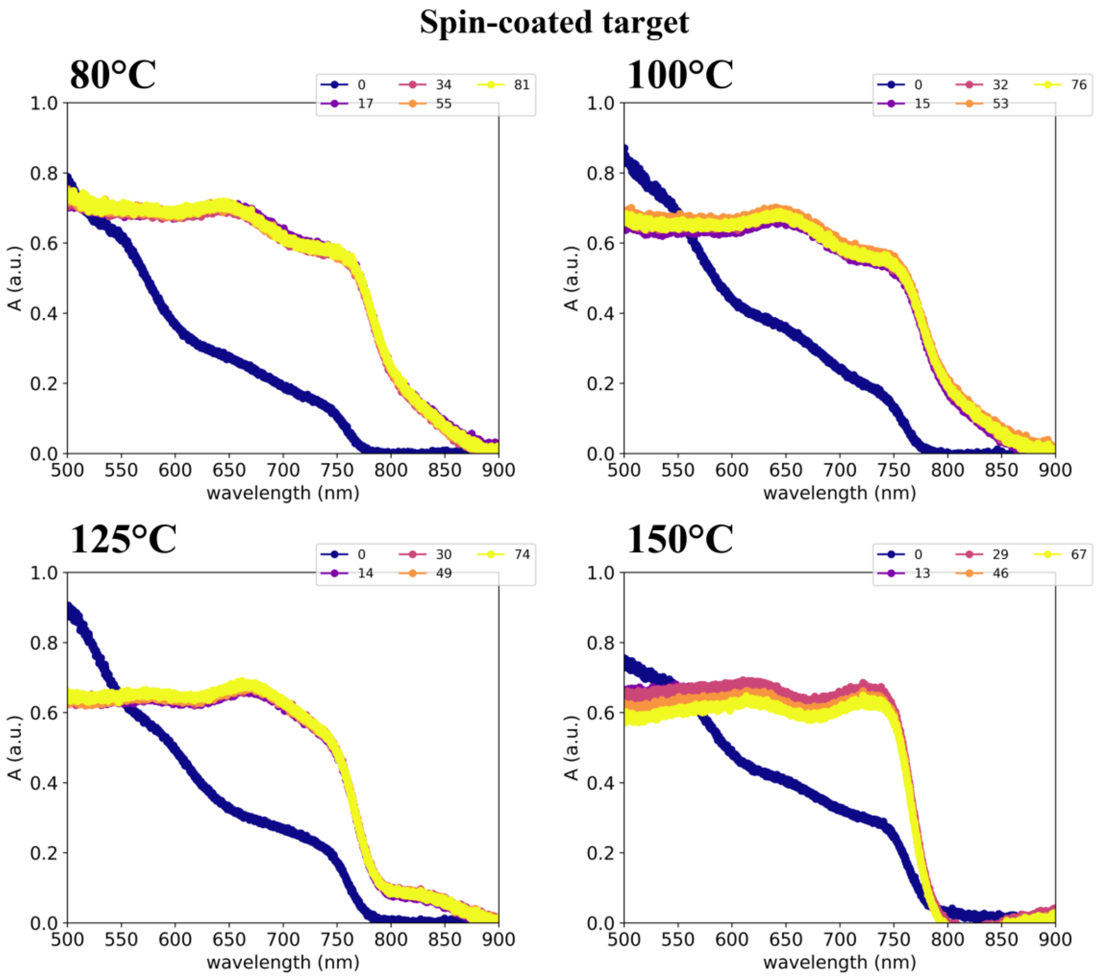

Figure S15 : Spectra derived from *in situ* optical measurements for spin-coated $Cs_{0.15}FA_{0.85}PbI_3$ films (target) annealed at different temperatures. The legend indicates the values of time (in s).

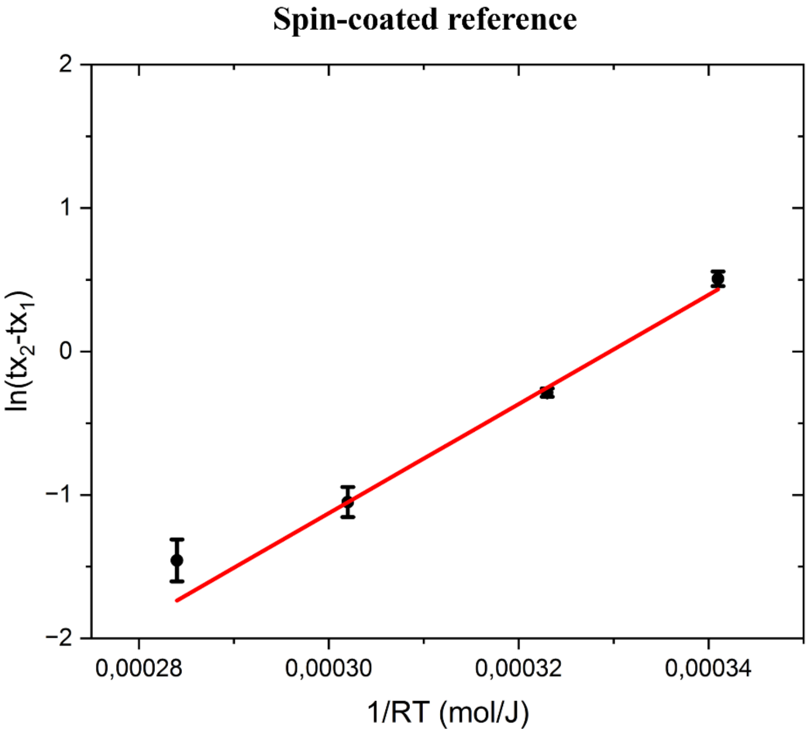

Figure S16 : Plots constructed from equation (1) to extract the activation energy E_A for spin-coated film $Cs_{0.15}FA_{0.85}PbI_3$ films (without TU additive in DMF/DMSO), slope of the line is 38 ± 4 kJ/mol.

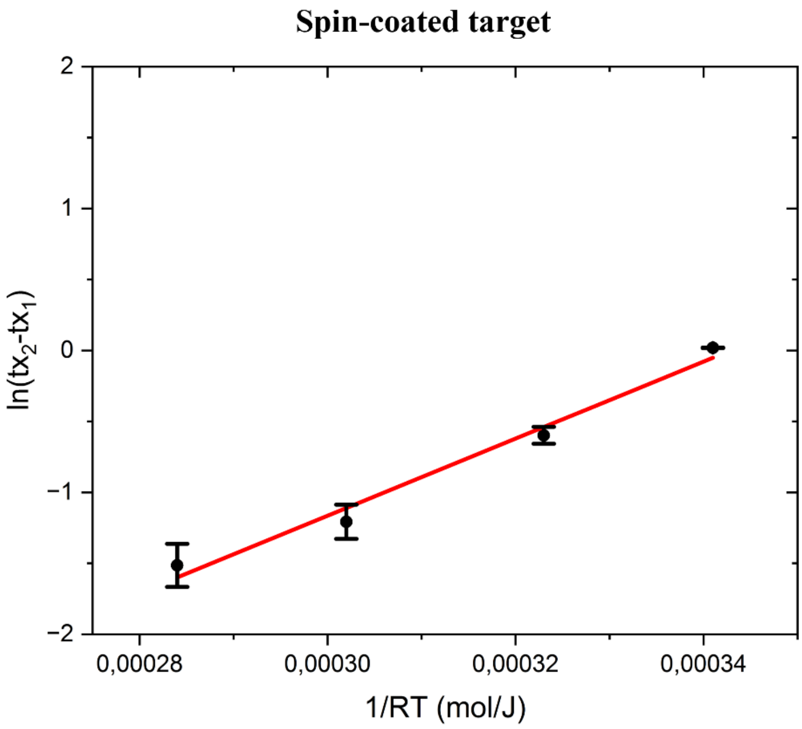

Figure S17 : Plots constructed from equation (1) to extract the activation energy E_A for spin-coated film (with TU additive in DMF/DMSO), slope of the line is 27 ± 3 kJ/mol.

Table S1 : Experimental parameters used for Mittemeijer's formula and obtained linear fit parameters.

Sample /Process	E_A (kJ/mol)	R^2
**Reference / blade-coated**	114 ± 21	0.93
**Target / blade-coated**	49 ± 6	0.97
**Reference / spin-coated**	38 ± 4	0.98
**Target / spin-coated**	27 ± 3	0.98

Table S2 : Kinetic parameters extracted from *in situ* optical measurements with the JMA kinetic model.

Sample / Process	Annealing temperature (°C)	K_0 (s^{-n})	K (s^{-n})	Avrami exponent n	R^2
**Reference / blade-coated**	80	$1.1 \times 10^{14} \pm 1 \times 10^{11}$	0.005	1.29 ± 0.002	0.99682
	100	$3.2 \times 10^{14} \pm 2 \times 10^{12}$	0.11	1.81 ± 0.02	0.99949
	125		1.1		0.99205
	150		7.8		0.96666
**Target / blade-coated**	80	$4.3 \times 10^{6} \pm 50720$	0.2	2.81 ± 0.03	0.9996
	100		0.5		0.99322
	125		1.4		0.99184
	150		3.3		0.95378

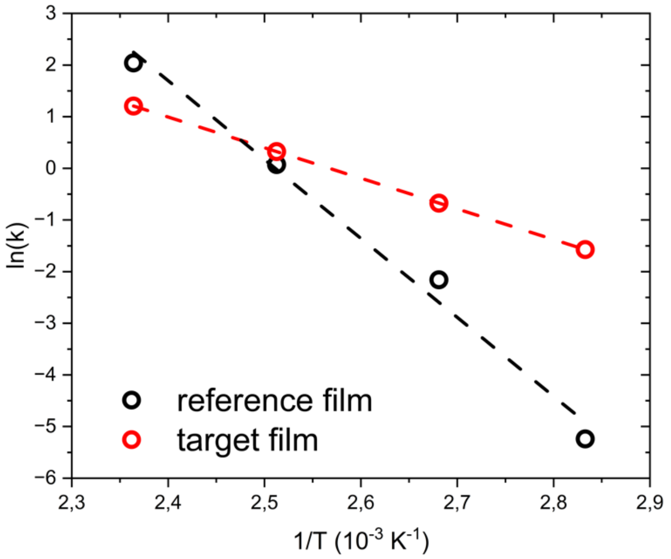

Figure S18 : Plots of ln(*k*) vs *1/T* for blade-coated perovskite films (reference and target).

(1) Qiu, S.; Majewski, M.; Dong, L.; Jang, D.; Corre, V. M. L.; Cerrillo, J. G.; Ronsin, O. J. J.; Yang, F.; Guo, F.; Zhang, K.; Lüer, L.; Harting, J.; Du, T.; Brabec, C. J.; Egelhaaf, H. In Situ Probing the Crystallization Kinetics in Gas□Quenching□Assisted Coating of Perovskite Films. *Adv. Energy Mater.* **2024**, *14* (10), 2303210.

(2) Rehermann, C. Exploring the Precursor-Process-Property Space in Metal Halide Perovskite Thin-Films. *PhD Diss.* **2020**, 1–150.

(3) Caiazzo, A.; Datta, K.; Jiang, J.; Gélvez-Rueda, M. C.; Li, J.; Ollearo, R.; Vicent-Luna, J. M.; Tao, S.; Grozema, F. C.; Wienk, M. M.; Janssen, R. A. J. Effect of Co-Solvents on the Crystallization and Phase Distribution of Mixed-Dimensional Perovskites. *Adv. Energy Mater.* **2021**, *11* (42), 2102144.
